# Nurses' Experiences of Caring for Children on Long‐Term Mechanical Ventilation

**DOI:** 10.1111/nicc.70330

**Published:** 2026-01-14

**Authors:** Annika Winblad von Walter, Veronica Lundberg, Sara Sonnemo, Anders Ringnér

**Affiliations:** ^1^ Department of Nursing Umeå University Umeå Sweden; ^2^ Department of Clinical Sciences, Paediatrics Umeå University Umeå Sweden; ^3^ Department of Health Sciences Karlstad University Karlstad Sweden

**Keywords:** children, long‐term home mechanical ventilation, paediatric nursing, parent–nurse relationship

## Abstract

**Background:**

The number of children with long‐term home mechanical ventilation (LTV) is increasing globally. Parents become experts on their children's needs and sometimes know more about LTV treatment than non‐specialist healthcare professionals. Nurses caring for LTV‐dependent children in intensive care settings adjust to a collaborative caregiving role; however, this interaction has not been described in general paediatric wards.

**Aim:**

To describe nurses' experiences of caring for children and adolescents with established LTV at general paediatric wards.

**Study Design:**

We conducted individual semi‐structured interviews with 15 participants and analysed the data using qualitative content analysis.

**Findings:**

The study resulted in three categories: ‘Building a relationship’, ‘Working hand in hand with the family’, and ‘Doing one's best despite the LTV’, interpreted into the theme ‘Working for reciprocal trust despite frail confidence’, which can be seen as foundational to nursing both the child and the family.

**Conclusions and Relevance to Clinical Practice:**

Reciprocal trust between families, nurses and the respiratory team could and should be established swiftly after admission with a discussion of each party's immediate responsibilities and back‐up roles.

## Impact Statements

- What is known about the topic
  ○ An increasing number of children and adolescents are treated with long‐term home mechanical ventilation (LTV).
  ○ Parents often perceive that their competence in LTV exceeds that of the healthcare professionals.
- What this paper adds
  ○ Nurses are aware of the conflict between their competence and parental demands.
  ○ By working hand in hand with the family, they strive to build a relationship in spite of a frail confidence.

## 1 Background

Throughout the world, the number of children and adolescents in need of long‐term home mechanical ventilation (LTV) has been increasing [1, 2]. These children can be vulnerable as they have complex health needs, often including several comorbidities such as chronic respiratory or lung diseases, weakness in respiratory muscles or central nervous system injury [2] or obstructive apnoea during sleep [3].

Different modalities, such as continuous positive airway pressure (CPAP) and bi‐level positive airway pressure (BiPAP), are used in LTV and can be administered in two ways. Non‐invasive ventilation is provided using a nasal, oral or face mask, whereas invasive ventilation means that the child is ventilated through a tracheostomy. LTV via a tracheostomy represents the most medically complicated group, as parents and caregivers then need training not only in the use of the ventilator but also in the complex care associated with the tracheostomy [2]. However, the focus for this paper is on non‐invasive ventilation as this constitutes the largest group of children with LTV [4].

Seriously ill children who are dependent on medical equipment are usually cared for in hospitals at the beginning of treatment. Here, the parents learn how to handle the equipment in order to allow the child to return home to their family [5]. In fact, parents often perceive that they provide more advanced care at home than nurses at non‐intensive care unit paediatric wards [6].

Parents of children with complex medical needs described becoming experts in their child's medical and care needs over time [5, 7, 8] and that they perceived that their knowledge regarding the child's care often exceeded that of the healthcare professionals (HCPs) [8]. Parents were able to identify and act early on their children's warning signs and various problems, and they sometimes felt distrustful of hospital care [8]. When the child needed medical care, parents felt that the HCPs at the children's ward had insufficient expertise to provide the advice and support the family needed [8, 9]. Families have a distrust of HCPs' experience and competence in the use of LTV and their own distrust of placing their children in hospital care [10]. However, parents also feared not being able to discern when or if their child's health and symptoms worsened and whether and when to seek healthcare for their child [8].

HCPs often depend on the parents' acquired knowledge and competence about their child's needs, while parents may feel that HCPs do not take advantage of their parental expertise. Being met with questions and condescension can cause parents anxiety, and being ignored can lead them to frustration and anger [5].

At the same time, HCPs may feel uncertain and worried about being or appearing incompetent during an already sick child's deteriorating health. HCPs feel stress caring for children treated with tracheostomy and LTV, especially during complications such as vomiting or accidental decannulation [9]. This lack of self‐confidence might also affect the quality of care and the relationship with the family [5].

Parental experiences of daily life with a child dependent on LTV are well described [11]. However, the experiences of parents and HCPs during the hospital admission of children with LTV are sparsely reported. In 2017, Denis‐Larocque and colleagues described the experiences of HCPs caring for LTV‐dependent children in ICU settings, which were characterised by nurses adjusting over time to a collaborative caregiving role in which they let go of some established ways of working [12]. This interaction, however, has not, to our knowledge, been described in general paediatric wards.

## 2 Aim

To describe nurses' experiences of caring for children and adolescents with established LTV at general paediatric wards.

## 3 Design and Methods

### 3.1 Design

This is a qualitative descriptive study using interviews analysed with qualitative content analysis according to Graneheim, Lindgren, and Lundman [13] and Graneheim and Lundman [14].

### 3.2 Context

The study was undertaken at a child and adolescent ward at a university hospital in Sweden. At this 18‐bed ward, patients with surgical, orthopaedic and paediatric problems are admitted both electively and as emergencies. The nursing staff at the ward consists of registered nurses and non‐registered assistant nurses who are assigned patients in pairs. The assignment is made informally for each shift by the nurses involved, depending on the total workload. Typically, a team of one registered nurse and one assistant nurse jointly are assigned four to five patients.

Swedish registered nurses complete a 3‐year bachelor's university degree in general nursing, which qualifies them to work also at paediatric wards. Most nurses subsequently undertake a 1‐year university programme in children's nursing to become specialists. Assistant nurses hold a vocational school diploma, with the option to pursue an additional 1‐year specialist diploma in children's nursing at a vocational school.

Children receiving LTV are regularly cared for at the ward, both at treatment startup and when hospitalisation is needed due to, for example, infections or surgical procedures. Some of these children also have personal care assistants (PCAs) funded by the government [15] to support their daily care needs, including managing the LTV equipment.

In close connection to the ward, a paediatric respiratory specialist team, comprising physicians, specialist nurses and physiotherapists, provides care for patients with LTV on a combined in‐ and outpatient basis. This team is one of four national units providing highly specialised care for children with rare pulmonary diseases.

### 3.3 Participants

We approached nursing staff (registered nurses and assistant nurses) with at least 2 years' experience in paediatric nursing. To ensure diversity within the sample, we selected participants strategically to represent different genders, professions and levels of work experience. When choosing between potential participants with similar backgrounds, we selected the one with the most experience in caring for children receiving LTV. After being informed about the study, those willing to participate gave informed consent and were interviewed.

### 3.4 Qualitative Interviews

Data were collected through individual, semi‐structured qualitative interviews [16]. An interview guide was constructed jointly by all authors and piloted in the first interview. No changes were made to the interview guide, and the pilot interview was included in the study. Most interviews were performed at the hospital. The interviews were recorded electronically and transcribed verbatim the same afternoon.

### 3.5 Qualitative Content Analysis

We read the text several times to get an overview of the content, then divided it into meaning units, each corresponding to one piece of content. The meaning units were condensed without losing the core message. All meaning units were assigned more logically abstract codes that we sorted according to similarities and differences into nine subcategories and three main categories. Finally, we identified an overarching theme. During the analysis, we authors discussed the data, codes and themes among ourselves and with research peers in seminars. The overall process was characterised by a focus shifting from specific details to the text and back again [13, 14]. To increase trustworthiness, the analysis was performed by three authors and then scrutinised by a fourth. The results were also discussed in seminars with research peers.

### 3.6 Ethics Statement

The study was performed according to the ethical principles of the Helsinki Declaration for Medical Research, thereby respecting participants' autonomy, integrity and confidentiality [17]. As no sensitive personal data were collected, an ethical review was not required by Swedish law ([18], p. 460). The study underwent review at the university department and was further approved by the head of the paediatric department (approval date: 01‐02‐2023). All participants were informed of the study, both in writing and verbally, including the voluntary nature of participation and their right to end their participation without explaining why. The participants consented to the study in writing. Each interview was assigned a code that could not be traced to the identity of the participant. All data were treated confidentially.

## 4 Findings

Of 45 nursing staff, 35 were eligible, 18 were approached and 15 chose to participate in the study and were each interviewed for between 11 and 22 min. For demographic data, see Table 1.

**TABLE 1:** Demographic data for participants.

Participants	*n* (15)	Median	Range
Gender			
Men	3		
Women	12		
Age		40.8	28–56
Profession			
Registered nurse	10		
Assistant nurse	5		
Specialist training in paediatrics^a^	8		
Years of experience		12	4–36

^a^ Specialist training in paediatrics consists of 1 year of advanced university studies for nurses and at a higher vocational school for assistant nurses.

An overarching theme, ‘Working for reciprocal trust despite frail confidence’, was identified as a common thread running throughout the results. Reciprocal trust was foundational for nursing both the child and the family, and it enabled the nurses to build relationships and work with both the family and their colleagues. Working towards this reciprocal trust, however, was hampered by limitations of the nurses' competence, support and resources concerning LTV, which weakened the nurses' confidence (Table 2).

**TABLE 2:** Overview of theme, categories and subcategories.

Working for reciprocal trust despite frail confidence	Building a relationship	Challenging first meetings
		Clear communication
		Varying support
	Working hand in hand with the family	High parental expectations
		Valuable joint knowledge
		Shared responsibilities
	Doing one's best despite the LTV	Ambivalent competence
		Unreliable backing
		Inadequate resources

### 4.1 Building a Relationship

Nurses worked to involve the whole family in the child's care and to establish a good relationship with the family. Clear communication facilitated the nursing, and giving various types of support reinforced the nurses' overall aim to be present and to instil confidence in the family.

#### 4.1.1 Challenging First Meetings

When a child with LTV was admitted, the nursing staff met not only the patient but the whole family. This first meeting was pivotal to how the relationship with the family evolved, as a bad first impression is hard to change:

You don't get a second chance to make a good impression. So, if you don't make a trustworthy impression the first time, then it will take a long time before you can be that person (RN 14).

The nurses stressed that they were honest about their own limitations in using the LTV equipment, but disclosed that they sometimes had to pretend to be in control to make the family feel confident:

If I have a feeling that, ‘Oh, I don't know if I can handle this’, that's what the family will see and feel or sense? (RN 14).

#### 4.1.2 Clear Communication

Nurses said that clear communication among nursing staff and parents facilitated the work, and they emphasised the importance of clarifying each person's responsibilities and sharing each other's expectations. They noted that the family is crucial in communications with the child, as often only the parents can interpret the child's signals:

What does your child mean when he moves his arm like that? Or this facial expression? Does it mean that it hurts? Or is he happy? Or this sound, does it mean anything? (RN 11).

The nurses identified barriers to communication, such as language disparity or stress, and without satisfactory communication, nurses and parents could become irritated and dissatisfied. Clear, perceptive communication, however, was considered to lead to good teamwork.

#### 4.1.3 Varying Support

Providing support is described as important for nurses, but the experience of what support is varies, and nurses reported that they seldom supported families directly with the mechanical ventilator. Still, they provided support in other ways. Emotional support comprised listening, being present, confirming the patients and their families and adapting to their needs. More tangible support included ensuring that the right materials were available, checking vital parameters and giving the family an opportunity to leave the bedside for some time.

Nurses also supported families by speaking up for them with other professionals. However, nurses found it difficult to provide support to unreceptive families, especially in stressful situations.

### 4.2 Working Hand in Hand With the Family

Nurses acknowledged that all involved parties strive towards the same goal, but in different ways. They highly valued the parents' acquired competence and always tried to utilise it in conjunction with their own professional competence. Measuring up to families' expectations was challenging, but nurses wished in the child's best interest for a clear and shared responsibility.

#### 4.2.1 High Parental Expectations

Nurses found it difficult to meet parents' expectations, and this created frustration and dissatisfaction for both parties. Nursing procedures had to be done exceptionally well, exceptionally quickly and almost perfectly according to the parents' preferences. This created stress and strain for the nurses, especially when they found other aspects of care more urgent than the parents did. Nurses also reported that parents, who often expected them to know and understand everything about the child and their LTV, were frequently annoyed when the nurses asked questions about things that parents believed the nurses should already know. Some nurses felt they were being tested and asked trick questions by the parents. However, nurses thought this could be rooted in parental worries and resignation:

I think they assume we know more than we do in some cases—like how everything works […], especially when it comes to CPAP and BiPAP (AN 1).

#### 4.2.2 Valuable Joint Knowledge

The nurses' goal was to work hand in hand with the parents, with everyone benefiting from each other's skills. Over time, parents gained broad and valuable knowledge about caring for an ill child in general, but also about managing home ventilators. Sometimes, this made parents ask advanced questions that were difficult for the nurses to answer.

In general, parents' acquired knowledge helped the nurses feel confident. The nurses took advantage of this by observing, asking questions and listening. They acknowledged that the parents knew their child best, and they referred to the parents as experts on their own child, the child's needs and the LTV.

However, nurses also noted that parents could perform procedures in a way that introduced risks for the child, and they found it difficult to challenge the parents in these situations. They concluded that they should definitely take advantage of the parents' knowledge, but still consider their own experience and professional competence. Furthermore, nurses were hesitant to argue with parents, as there are often several ways to perform a procedure while still keeping it safe for the child:

… you don't just naturally go in and make the precise hand grips that… the family or parents expect you to make… and it's also partly based on the fact that there isn't just one way to do it right… (RN 14).

#### 4.2.3 Shared Responsibilities

Nurses had different views on how to share responsibility for the child's care. Some nurses said that the parents should be responsible for the ventilator as they were more experienced with it. Other nurses believed that responsibility should lie with the HCPs:

After all, the responsibility lies with us when they are hospitalized… It's not the parents who should have all the responsibility, it's us! (RN 2).

When the sharing of responsibility was unclear, nurses saw a risk that the child could be harmed.

### 4.3 Doing One's Best Despite the LTV

According to nurses, nursing patients with LTV was complex and required several resources that might not always be available. Possessing the appropriate skills and having competent colleagues available were key to nurses' confidence in this situation. Nurses felt somewhat safe, but also often insecure, in caring for patients with LTV; therefore, they wished for more education on the subject to bolster their confidence and improve their care of the patients and their relationship with the families.

#### 4.3.1 Ambivalent Competence

In general, nurses felt confident in managing common CPAP and BiPAP devices, and most nurses had undergone basic training in how to use them.

Nevertheless, the same nurses reported uncertainty about how to operate familiar, but especially unfamiliar, equipment and invasive ventilation via tracheostomy:

If it's a familiar device, it's better, since we have that device [on the ward]. But if it's not, it's very unsafe. Then you have zero knowledge. And if it's a home respirator, you're actually completely in trouble, because we don't have any respirator training (RN12).

This ambivalence was due to knowledge gaps, high expectations from parents and a lack of experience with children using invasive respiratory support:

I feel confident but still unsecure! Because I don't have all the skills that I feel I need (RN11).

The knowledge gaps made the nurses feel that they had no control and were forced to redirect their nursing interventions from operating the device to monitoring vital signs.

#### 4.3.2 Unreliable Backing

Increased knowledge of ventilators and competent colleagues who were used to the device gave nurses increased confidence in their professional role, as did the opportunity to consult the paediatric respiratory specialist team at the department:

I feel confident when there are staff around me who know. So, in that way I'm secure (RN3).

Other factors that increased nurses' confidence were clear routines, a confident working group and mental preparation for potential emergencies. However, when there was a mismatch between demands and competence, nurses felt stressed and the child's safety was at risk.

Virtually all children with home ventilators had PCAs who accompanied the family when they were admitted to the hospital. The nurses felt more confident when they saw that the PCA knew the child well and possessed the necessary knowledge. However, when PCAs did not have the required skills, nurses felt anxious. They needed to double‐check what the PCAs were doing, which could be stressful.

#### 4.3.3 Inadequate Resources

The nurses perceived that patients with ventilators required more time than otherwise healthy children, as they often had significant and unpredictable needs and could quickly deteriorate. They found this concerning, especially if the patient acuity level on the ward did not match the staffing.

Nurses reported that the health care resources were not always sufficient to meet the needs of the children. It was not always possible to assign staff to be in the room with the child when needed. The participants experienced that critically ill children with LTV often remained in the ward for too long before they were moved to the intensive care unit:

I ran to the ICU with a patient, who I thought would die on the way. I think that was in the last minute… when we ran there… (RN12).

Nurses said it inspired a sense of calm when the child was transferred to the appropriate level of care in time, and that it felt good when the child recovered.

## 5 Discussion

The aim of this study was to describe nurses' experiences of caring for children and adolescents with established LTV at general paediatric wards. The results show nurses' experiences illustrated by the categories ‘Building a relationship’, ‘Working hand in hand with the family’ and ‘Doing one's best despite the LTV’, all collected under the theme ‘Working for reciprocal trust in spite of frail confidence’.

Nurses in this study strove to build working relationships with the families. They considered clear communication as a fundamental, but sometimes difficult, part of this relationship building. Poor communication could cause irritation and dissatisfaction. Building relationships and working closely hand in hand with patients and families has long been considered essential in both general nursing [19, 20] and contemporary child‐centred care [21]. The need for clear communication with nurses has also been emphasised by parents of children with complex conditions, such as dependence on LTV. Parents also value nurses' acknowledgement of their experiences and knowledge of the child's caring needs [6]. A mutual understanding of the plan of care is considered a good outcome of the communication process [22]. Nurses in the ICU report having to engage in constant negotiation about the boundaries of responsibility [12]. Talking explicitly about the division of work and responsibilities around the LTV treatment would probably improve confidence in both nurses and parents.

When nurses in our study lacked confidence in managing the clinical and technical challenges of using LTV equipment, problems arose that influenced their ability to work well with the family. Technical know‐how is considered one of several nursing competencies [23], and in our study, the nurses felt less competent when they were not able to match the expectations of their patients' parents. This made the nurses feel that parents' trust in them was diminished. They also felt that parents tested them on their knowledge, which is supported by other studies describing how parents safeguarded their children by testing the nurses [6, 22].

The nurses in this study adopted different strategies when they lacked confidence in managing LTV devices. Having an opportunity to consult colleagues who were familiar with the device and treatment, such as the paediatric respiratory team, was essential in these situations. Lack of training and experience in a clinical area has been shown to contribute to uncertainty [24].

Parents have also described the high responsibility and pressure they felt when their child was introduced to LTV [25]. These concerns are understandable, since there are significant safety issues that need to be considered, especially in children on invasive LTV via tracheostomy tube, who are a complex patient group requiring close monitoring [26]. The children's respiratory function may be affected as LTV involves technical complexity, and tracheostomy‐related complications may also occur [27]. The key challenge to the nurses in our paper appears to be in caring for an already severely ill child, treated with LTV in the home, who deteriorates and needs hospitalisation. The parents should be able to hand over the responsibility for the LTV to the HCPs, but the treatment provided at home often requires familiarity with LTV beyond what could be expected from a general nurse at a diverse children's ward.

It is probably unrealistic to expect these nurses to be experts on how to operate all available models of LTV equipment even better than a parent who uses one specific model on a daily basis. Still, when caring for children with LTV, nurses must have in‐depth knowledge about respiratory physiology, the principles of LTV and the risk of complications in addition to their general competence in nursing a severely ill child. The nurses and the parents can then bring different kinds of knowledge to the situation [28]. The parents' knowledge is situation‐based and mostly specific to a certain child, but the nurses' competence is more generic and can safeguard and predict changes in situations other than the child's ordinary health status. How these complementary forms of knowledge interact is still under‐reported in research. Having the opportunity to consult the paediatric respiratory team, who can provide expert knowledge on specific LTV equipment, is crucial to providing safe care for the child and boosting confidence and safety in the nurse caring for the child. Also, simulating paediatric emergencies has been shown to decrease anxiety and increase confidence in nurses [29]. Such a strategy in paediatric care, especially involving LTV, may be effective in increasing nurses' confidence in caring for these patients and their families. At the end of the day, this is needed to give high‐quality care to patients as well as being fair to the nursing staff.

In the Swedish healthcare system, parents are encouraged to take an active role in their child's medical care [30], for example managing a tracheostomy at home. As reported in this study, parents may occasionally perform these procedures in ways that do not strictly follow established protocols. However, parents caring for chronically ill children often need to be innovative, adapting procedures to the child's needs and the home environment [31]. This, in turn, requires nurses to exercise professional judgement to determine whether such adaptations constitute a risk‐inducing deviation or simply an alternative, safe way of performing the procedure.

Nurses in this study had different opinions about who should be responsible for what. Most nurse participants preferred parents to take the main responsibility for the LTV as parents had the most knowledge about the specific child's care, treatment and LTV device. Parents have also reported that they often prefer to provide care themselves in a manner familiar to the child [6], which is consistent with the tradition within the Swedish healthcare system [30]. The question of who should be responsible for what is nevertheless disputed and can cause conflicts between parents and HCPs [7]. At one extreme, delegating most tasks to parents may amount to an abdication of professional responsibility, whereas at the other extreme, denying parents the opportunity to care for their child reflects a form of paternalism that disregards their wishes and knowledge of the child. This clearly necessitates compromises from both parties. If nurses and parents take advantage of each other's skills and have a clear division of responsibilities, their cooperation can be improved and the goal of working hand in hand can be achieved. If this division of responsibilities is negotiated, made explicit and documented early after admission, it could create confidence in both parties. Parents and nurses can complement each other dynamically by collaborating on initiated measures and supporting and helping each other [32]. However, the final responsibility for the child's health is with HCPs when the child is hospitalised.

## 6 Limitations

Although this study was performed at only one ward at a tertiary university hospital, most nurses in general children's wards have the same educational background and understanding of LTV, which adds to the generalizability of the data. The participants varied by work experience and age, but the proportion of male nurses was low, mirroring the continuing demographics of HCPs.

## 7 Implications for Practice and Further Research

Clearly and quickly establishing the different responsibilities of families and nurses respectively as soon as possible after admission could be helpful to diminish role confusion in this context. Further, nurses caring for this frail and complex patient group need to be provided with adequate support and education from specialist teams. Such models should be tested in clinical trials to provide better evidence. Finally, future research should focus on the parental perspective to contribute to an increased mutual understanding of the different caregiving roles.

## 8 Conclusion

This study provides a nuanced understanding of nurses' experiences in caring for children with LTV and their families. Meeting knowledgeable parents with high expectations, their own lack of experience of LTV treatment and insufficient support are dilemmas and difficulties that nurses face when striving to establish a reciprocal trust in spite of frail confidence.

## Funding

The authors have nothing to report.

## Consent

All participants were informed verbally and in writing about the study, the voluntary nature of their participation and their right to end their participation without explaining why. The participants gave their written consent to participate.

## Conflicts of Interest

The authors declare no conflicts of interest.

## Data Availability

The data that support the findings of this study are available from the corresponding author upon reasonable request.
